# Hurry and Worry

**Published:** 1885-04

**Authors:** 


### SELECTIONS.

### Hurry and Worry.

It is not "overwork," but worry, that kills. Our men of brain might do a great deal more than they do, if only they were less feverish in their haste, less harassed by worry, and lest wasteful of energy. We are all in too much of a hurry in what we do. We have too many irons in the fire, too much business on hand at the same instant, and are far too energetic in our endeavors. With deliberation, calmness, and such reserve of strength as results from perfect restraint, a man may do an infinity of work without either trouble or energy.

The system of breathless haste and eager anxiety is rapidly undermining the human constitution. We are impatient for results. Statesmen and politicians are kept on the strain of sustained attention, and their brains are for many hours of the twenty-four in a state of ferment. The brains of speculators on the Stock Exchange, and even the brains of merchants in their private rooms, are equally taxed in the same way. All classes of the community share the turmoil. The period is one of brainworking impetuosity; of hurry, worry, and waste—the waste of cerebral energy and nerve force. The higher nerve-centres are kept incessantly at work, and become, as it were, overheated, so that it is impossible they should quiet or cool down in the brief period of time allotted to repose. Too often they do not rest even in sleep. The brain only dozes instead of sleeps, and as a result there are dreams of the recent day's work—that infallible symptom of impending mischief. The only marvel is that, looking to the utterly unphysiological character of our mental and nervous habits of work, the number of sudden failures is not greater than it is, and that we have not a larger percentage of brain mortality to deplore.—*London Lancet.*

